# Is Smoking Associated with Carpal Tunnel Syndrome? A Meta-Analysis

**DOI:** 10.3390/healthcare10101988

**Published:** 2022-10-11

**Authors:** Kaisa Lampainen, Sina Hulkkonen, Jorma Ryhänen, Stefania Curti, Rahman Shiri

**Affiliations:** 1 Department of Hand Surgery, Helsinki University Hospital and University of Helsinki, 00014 Helsinki, Finland; 2 Department of Hand Surgery, Seinäjoki Central Hospital, 60220 Seinäjoki, Finland; 3 Department of Medical and Surgical Sciences, University of Bologna, 40138 Bologna, Italy; 4 Finnish Institute of Occupational Health, 00250 Helsinki, Finland

**Keywords:** median nerve, median neuropathy, systematic review, tobacco, lifestyle

## Abstract

To date, the role of smoking in carpal tunnel syndrome (CTS) is unclear. The aim of this systematic review and meta-analysis was to assess the association between smoking and CTS. The literature searches were conducted in PubMed, Embase, and Scopus, from inception until October 2021. Three reviewers screened the titles, abstracts, and full-text articles and evaluated the methodological quality of the included studies. A random-effects meta-analysis was used, and heterogeneity across studies was examined using I^2^ statistic. A total of 31 (13 cross-sectional, 10 case-control, and 8 cohort) studies were qualified for meta-analysis. In a meta-analysis of cohort studies, the risk of CTS did not differ between current and never smokers (pooled hazard ratio (HR) 1.09, 95% CI 0.84–1.43), current and past/never smokers (HR 1.07, 95% CI 0.94–1.23), and past and never smokers (HR 1.12, 95% CI 0.83–1.49). Furthermore, a meta-analysis of case control studies found no difference in the risk of CTS between current and never smokers (pooled odds ratio (OR) 0.92, 95% CI 0.56–1.53), current and past/never smokers (OR 1.10, 95% CI 0.51–2.36), and past and never smokers (OR 0.91, 95% CI 0.59–1.39). However, a meta-analysis of cross-sectional studies showed the associations of ever (OR 1.36, 95% CI 1.08–1.72) and current smoking (OR 1.52, 95% CI 1.11–2.09) with CTS. However, the association between ever smoking and CTS disappeared after limiting the meta-analysis to higher quality studies or after adjusting for publication bias. The association between current smoking and CTS also attenuated after limiting the meta-analysis to studies that confirmed CTS by a nerve conduction study or studies with low attrition bias. This meta-analysis does not support an association between smoking and CTS. The association between smoking and CTS observed in cross-sectional studies could be due to biases and/or confounding factors.

## 1. Introduction

Compression of the median nerve at the carpal tunnel, known as carpal tunnel syndrome (CTS), is the most common entrapment neuropathy of the upper extremity [1,2,3]. The incidence of CTS varies between 88 and 105 cases per 100,000 person-years among men and between 193 and 232 cases per 100,000 person-years among women [4,5,6]. The etiology of CTS is multifactorial; often, both occupational and personal risk factors are involved. Its known risk factors include female gender, excess body mass, diabetes mellitus, rheumatoid arthritis, and thyroid disease [7,8,9,10,11,12,13]. Manual workers are at higher risk of CTS than non-manual workers [14]. Genetic factors might also play a role in CTS [15].

Smoking is a major health concern [16]. To date, the role of smoking in CTS remains unclear. Cigarette smoking is associated with reduced blood supply, oxidative stress, and systemic inflammation, which might impair the peripheral nerves and make them more vulnerable to compression neuropathies [17,18]. As found to be a neuroteratogen in animal models, smoking may also increase the risk of median nerve damage through toxic effects [19]. Smoking was also associated with ulnar neuropathy at the elbow [20].

An earlier meta-analysis regarding the association between smoking and CTS, published in 2014 by Pourmemari and his colleagues, reported inconclusive results [21]. That meta-analysis found an association between current smoking and CTS in cross-sectional studies, but not in case control or cohort studies. Only three prospective cohort studies were included in that meta-analysis, and none of those was a high-quality cohort study [22,23,24]. Since the previous meta-analysis, multiple studies on the role of smoking in CTS have been published, including three large, population-based longitudinal studies [25,26,27].

The aim of this systematic review and meta-analysis was to determine whether smoking is associated with CTS.

## 2. Methods

We developed the protocol of this systematic review and meta-analysis according to the PRISMA guidelines [28]. We retained the studies included in the earlier meta-analysis by Pourmemari and colleagues [21] and performed literature searches from inception to October 2021. The study protocol is registered in PROSPERO (registration no. 347845).

### 2.1. Search Strategy

Literature searches were conducted in PubMed, Embase, and Scopus, from their inception until October 2021. We used a combination of MeSH terms (in PubMed), Emtree terms (in Embase), and text words (Table 1). The search strings for PubMed and Embase were similar to those used in the previous meta-analysis [21]. We also manually searched the reference lists of the included studies to locate the additional studies. We included all languages and excluded case reports, letters, editorials, guidelines, and reviews.

### 2.2. Inclusion and Exclusion Criteria

Three reviewers (K.L, S.H., and R.S.) independently screened the titles and abstracts of the references retrieved. Both population- and hospital-based case-control, cross-sectional, and cohort studies that reported quantitative results for the association between smoking and CTS symptoms confirmed by nerve conduction studies or clinical signs were included in the meta-analysis. Studies conducted among volunteers and CTS patients without a control group were excluded. Moreover, studies defined CTS based on self-reports, studies defined CTS by symptoms only, or nerve conduction studies only were excluded. Lastly, studies conducted among pregnant women, patients undergoing dialysis, or among patients with toxic oil syndrome were excluded from the review. Disagreements between the reviewers were resolved through discussion.

#### 2.2.1. Data Extraction

Characteristics of the included studies and quantitative data were extracted by two reviewers (S.H. and K.L.) and checked by a third reviewer (R.S.). The following characteristics of the included studies were extracted: study population, age and gender distribution, sample size, smoking, outcome assessment, summary results, and adjustment for confounding factors.

#### 2.2.2. Quality Assessment

Three reviewers (K.L., S.H., and R.S.) independently appraised the risk of bias of included studies. For methodological quality assessment, we used a checklist adapted from the Effective Public Health Practice Project tool [28]. We rated the quality of each study, according to five sources of bias: selection, performance, detection, confounding factors, and attrition (Appendix A Table A1). Disagreements between reviewers were resolved through discussion.

#### 2.2.3. Statistical Analysis

Odds ratio for cross-sectional and case-control studies and risk ratio for prospective cohort studies were estimated for those studies reporting descriptive results, such as the number of CTS cases in smokers and non-smokers or number of smokers in CTS cases and controls. The Woolf confidence interval was calculated for the estimated odds ratios [29]. Since the prevalence of CTS is less than 5%, we did not convert odds ratios to risk ratios for the meta-analysis of prospective cohort studies. With a prevalence of less than 5%, the odds ratio is identical to risk ratio. A random-effects meta-analysis was used to combine the estimates of studies, and the I^2^ statistic was used to assess the presence of heterogeneity across the studies [30,31]. Subgroup analyses were conducted with regard to methodological quality of included studies. A funnel plot was used for exploring publication bias, and Egger’s regression test was used for examining funnel plot asymmetry. Due to small number of studies included in the meta-analyses, only presence or absence of bias in one quality domain was used for subgroup analysis. Furthermore, the trim and fill method was used to adjust for missing studies, due to publication bias [32,33]. Stata version 17 (StataCorp LP, College Station, TX, USA) was used for the meta-analyses.

## 3. Results

**Figure 1:**
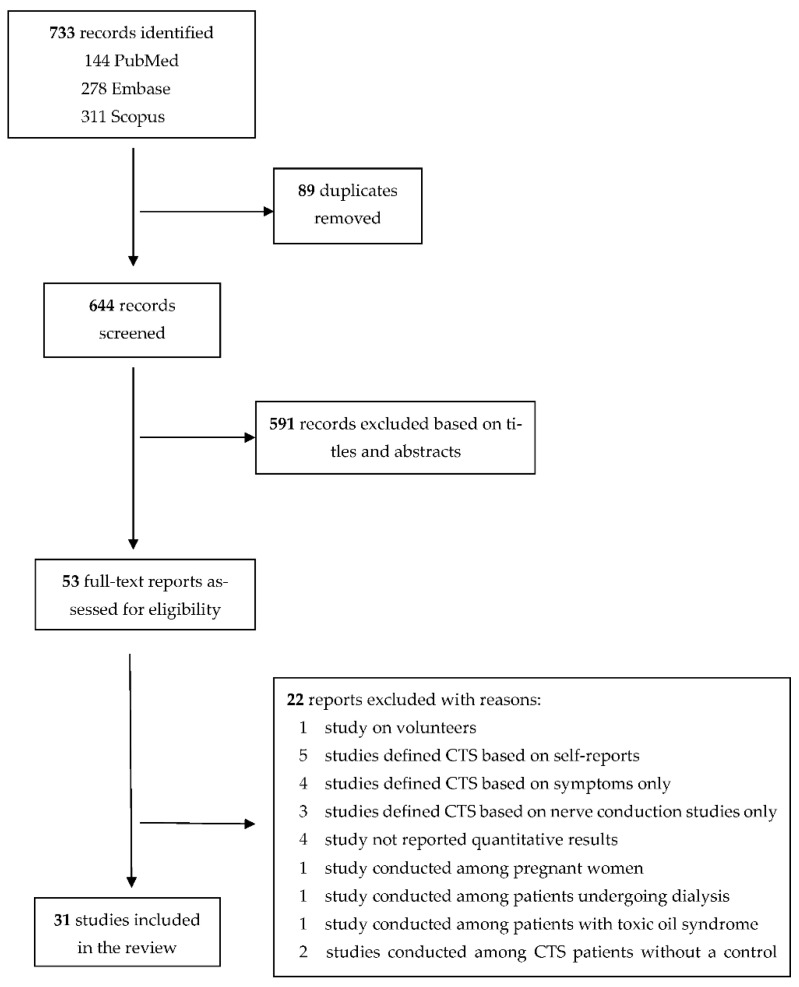
PRISMA flow diagram of study selection.

A total of 733 records were identified. After removing duplicates, 644 were screened. Of these, 591 were excluded based on titles and abstracts, and 53 full-text reports were assessed for eligibility. Of these, 22 reports were excluded with reasons (Figure 1). Finally, 31 studies, consisting of 13 cross-sectional studies [10,34,35,36,37,38,39,40,41,42,43,44,45], 10 case-control studies [11,46,47,48,49,50,51,52,53,54], and 8 cohort studies [22,24,25,26,27,55,56,57], were included in the meta-analysis. The characteristics and quality of the included studies are reported in Appendix A Table A2, Table A3 and Table A4.

**Figure 2:**
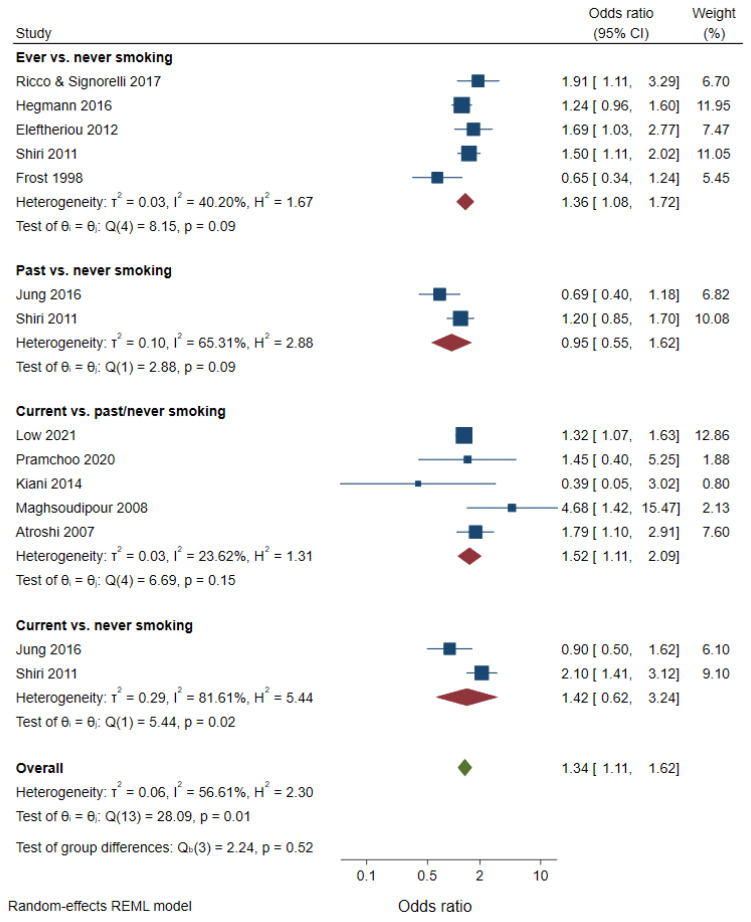
Meta-analysis of cross-sectional studies on smoking and CTS.

A meta-analysis of cross-sectional studies showed a higher prevalence of CTS among ever smokers, compared with never smokers (OR 1.36, 95% CI 1.08–1.72, Figure 2), as well as among current smokers, compared with past/never smokers (OR 1.52, 95% CI 1.11–2.09). Of note, a small (*n* = 379) cross-sectional study examined the association between number of packs per years smoked and CTS, but no association was found [44]. In the sensitivity analyses, the association between ever smoking and CTS disappeared after limiting the meta-analysis to higher quality studies or adjusting for publication bias (Table 2). The association between current smoking and CTS was not due to publication bias, selection bias, or confounding factors. The association did not remain statistically significant when the meta-analysis was limited to the studies with CTS confirmed by a nerve conduction study or to those studies with low attrition bias.

**Figure 3:**
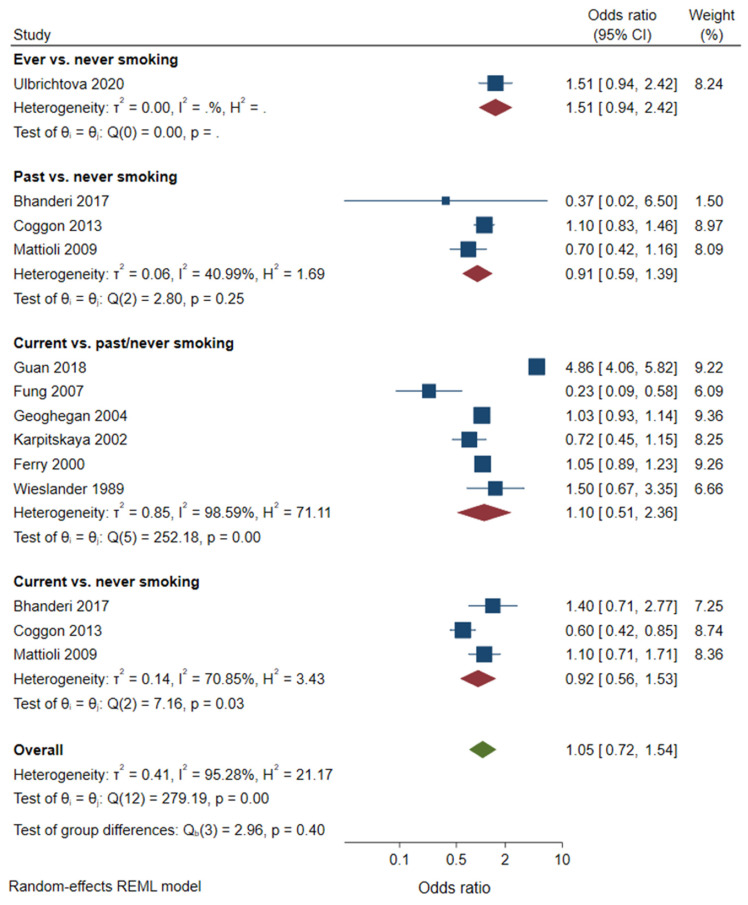
Meta-analysis of case-control studies on smoking and CTS.

A meta-analysis of case control studies showed no associations of ever, past, and current smoking with CTS (Figure 3). The pooled OR was 0.92 (95% CI 0.56–1.53, three studies) for current smoking, compared with never smoking, 1.10 (95% CI 0.51–2.36, six studies) for current smoking, compared with past/never smoking, and 0.91 (95% CI 0.59–1.39, three studies) for past smoking, compared with never smoking.

**Figure 4:**
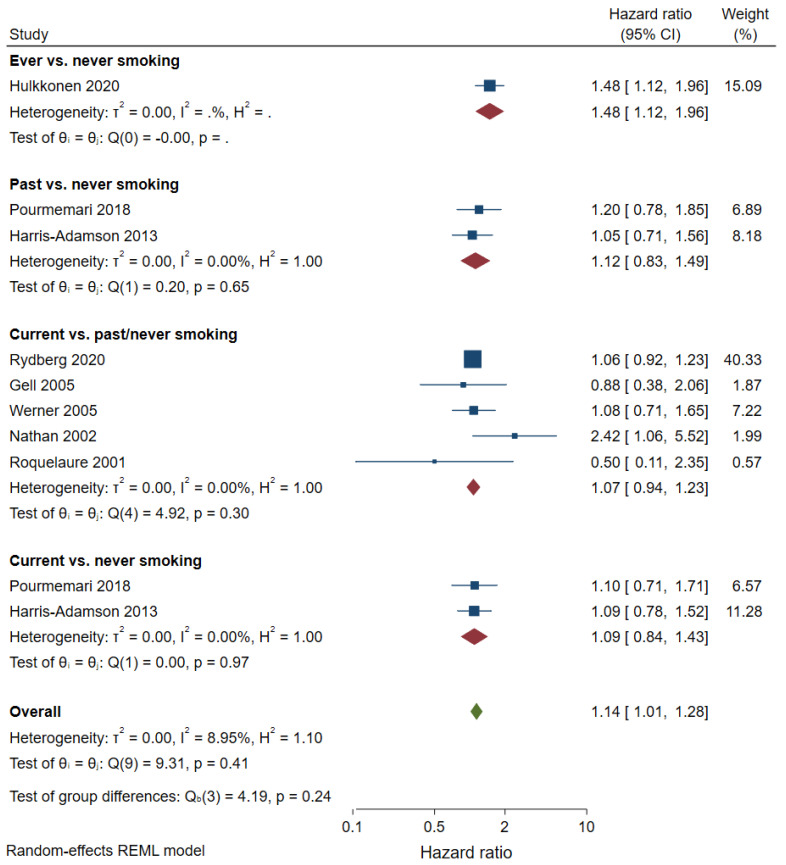
Meta-analysis of prospective cohort studies on smoking and CTS.

A meta-analysis of prospective cohort studies showed that the incidence of CTS does not differ between current and never smokers (hazard ratio [HR] 1.09, 95% CI 0.84–1.43, two studies, Figure 4), current and past/never smokers (HR 1.07, 95% CI 0.94–1.23, five studies), and past and never smokers (HR 1.12, 95% CI 0.83–1.49, two studies). Only one cohort study compared ever smokers with never smokers (HR 1.48, CI 1.12–1.96). One prospective cohort study (*n* = 8703) explored the association of the number of pack-years smoked and hospitalization for CTS [58]. Among men, pack-years > 10 was associated with hospitalization for CTS but not pack-years ≤ 10, after adjustment for body mass index, socioeconomic status, and diabetes. Among women, both pack-years ≤ 10 and pack-years > 10 were associated with hospitalization for CTS.

## 4. Discussion

In this meta-analysis, we found no association between smoking and CTS in case control or cohort studies. Only a meta-analysis of cross-sectional studies showed an association between smoking and CTS. The results of the current meta-analysis are consistent with those of a previous systematic review and meta-analysis of studies published up to 2014 [21]. Limiting the meta-analysis of cross-sectional studies to higher quality research did not support an association between smoking and CTS.

The lack of uniformity in using a comparison group for current smoking across the included studies reduced the statistical power of this meta-analysis. A meta-analysis of cross-sectional studies did not show a significant difference in the prevalence of CTS between current and never smokers, but showed a significant difference between current and past/never smokers. Furthermore, most of the studies included in the current meta-analysis did not assess the association between the number of cigarettes smoked per day and CTS.

Recent studies have identified the relationship between workload factors and CTS [26,59,60]. Occupational biomechanical factors, such as forceful handgrip, repetitive wrist extension and flexion, extreme wrist postures, and use of vibratory tools, play a role in the causation of CTS [26,59,60,61]. In this meta-analysis, we found an association between smoking and CTS in cross-sectional studies; however, some of these studies did not adjust their estimates for work-related factors. It would be worth noting that blue-collar workers are more likely to smoke [62]. It is possible that the association between smoking and CTS in cross-sectional studies is confounded by work-related factors. In the sensitivity analysis of cross-sectional studies, the association between smoking and CTS was attenuated after limiting the meta-analysis to higher quality studies. It is likely that the association between CTS and smoking observed in cross-sectional studies is not a true association. It may be due to biases and/or confounding factors.

With respect to the meta-analysis of case control studies, we found no association of ever, past, or current smoking with CTS. It is possible that hospital-based controls have influenced the outcomes, as most of the included studies in this meta-analysis used hospital-based controls [11,47,48,49,51,52]. Only one case control study used both population- and hospital-based control groups [54]. In particular, there was a higher proportion of current smokers among hospital controls (29%) than population-based controls (19%). Hospital-based controls are likely to have other latent or undiagnosed diseases. Many studies have shown that the prevalence of CTS is significantly higher, for example, among patients with postmastectomy lymphedema or chronic hemodialysis than among the general population [63,64,65,66]. Using hospital patients as a control group may underestimate the true association between smoking and CTS.

The studies included in the current meta-analysis had some limitations. Smoking was assessed subjectively, rather than objectively, which makes it prone to recall bias. Study participants may underreport their tobacco consumption [67]. Another possible explanation for underreporting is that smoking tends to be a habitual and almost unconscious habit [68]. Some of the included studies did not control their estimates for the known risk factors of CTS. The observed association in cross-sectional studies can partly be due to confounding factors. Furthermore, most of the included studies did not collect data on the number of cigarettes smoked per day, number of years spent smoking, and duration of smoking cessation. Thus, we were not able to explore a dose-response relationship between smoking and CTS.

## 5. Conclusions

In this meta-analysis, we found no association between smoking and CTS in the meta-analyses of case control and cohort studies. Smoking was associated with CTS only in a meta-analysis of cross-sectional studies. However, limiting the meta-analysis to higher quality cross-sectional studies did not support an association between smoking and CTS. It is likely that the association between smoking and CTS observed in cross-sectional studies is not a true association.

## Figures and Tables

**Table 1:** PubMed, Embase, and Scopus searches, conducted on 2 October 2021.

Search	Query	No of Items Found
**PubMed**		
	(carpal tunnel[tiab] OR carpal tunnel syndrome[MeSH] OR median nerve[tiab] OR median neuropathy[tiab]) AND (smok * OR tobacco[tiab] OR cigar * OR life-style OR lifestyle)	144
	**Embase**	
	(‘carpal tunnel syndrome’:ab,ti OR ‘median nerve compression’:ab,ti OR ‘median nerve’:ab,ti OR ‘carpal tunnel syndrome’/exp OR ‘median nerve compression’/exp OR ‘median nerve injury’/exp) AND (smok *:ab,ti OR cigar *:ab,ti OR ‘smoking’/exp OR ‘cigarette’/exp OR ‘cigar’/exp OR ‘tobacco’/exp OR tobacco:ab,ti OR lifestyle:ab,ti OR life-style:ab,ti)	278
	**Scopus**	
	(carpal tunnel OR median nerve OR median neuropathy) AND (smok * OR tobacco OR cigar * OR life-style OR lifestyle)	311

**Table 2:** Sensitivity analyses of cross-sectional studies on the associations of ever and current smoking with CTS, according to methodological quality of included studies and adjustment for publication bias.

Risk of Bias	Ever Smoking				Current Smoking			
	No. of Studies	OR	95% CI	I^2^ (%)	No. of Studies	OR	95% CI	I^2^ (%)
Overall	5	1.36	1.08–1.72	40	7	1.54	1.13–2.09	49
Adjustment for publication bias	6	1.28	0.99–1.65		7	1.54	1.13–2.09	
Selection bias								
Low	1	1.50	1.11–2.02	-	2	1.97	1.45–2.68	0
Moderate	3	1.16	0.73–1.85	69	4	1.39	0.87–2.21	50
High	1	1.91	1.11–3.29	-	1	0.39	0.05–3.02	-
Confounding								
Low	2	1.04	0.46–2.34	81	2	2.55	1.30–5.00	36
Moderate	2	1.34	1.03–1.75	16	3	1.40	1.13–1.75	6
High	1	1.91	1.11–3.29	-	2	0.84	0.48–1.49	0
Detection bias								
Low	3	1.19	0.69–2.04	74	4	1.63	0.89–3.00	58
Moderate	2	1.55	1.20–2.00	0	3	1.52	0.97–2.36	62
Attrition bias								
Low	3	1.31	c	75	5	1.48	0.82–2.65	52
Moderate	2	1.34	1.11–1.63	0	2	1.61	1.03–2.53	76

## Data Availability

Not applicable.

## Appendix A

**Table A1:** Quality assessment.

Type of Domain	Criteria Definition	Classification (Potential for Bias)
Selection bias	Sampling method of the study population, representativeness, response rate, difference between responders and non-responders, investigation, and control of variables, in case of difference between responders and non-responders	Weak: Target population defined as representative of the general population or subgroup of the general population (specific age group, women, men, specific geographic area, and specific occupational group), and response rate is above 80%. Moderate: Target population defined as somewhat representative of the general population, a restricted subgroup of the general population, response rate 60–79%. Strong: Target population defined as “self-referred”/volunteers, response rate less than 60%.
Performance bias	Valid and reliable assessment of exposure Assessors blinded for outcome status	Weak: Smoking status was defined as never, past, and current smokers. Information on the number of cigarettes smoked per day or number of pack-years smoked. The assessors of smoking status blinded towards the outcome. Moderate: Smoking status was defined as never, past, and current smokers. No information on the number of cigarettes smoked/day or number of pack-years smoked. Strong: A dichotomous question was used, and never-smokers or current smokers were not recognized from past smokers, assessors not blinded to outcome status.
Detection bias	Clear definition of outcome Standard method for outcome assessment Assessor of outcome blinded to exposure status	Weak: The outcome was defined by clinical diagnosis and nerve conduction studies. Moderate: The outcome was defined by clinical diagnosis only. Strong: Self-reported outcome, assessors not blinded to exposure status.
Confounding	Matching Stratification Statistical analysis	Weak: Considered confounders and controlled for 80–100% of confounders. Moderate: Considered confounders and controlled for 60–79%. Strong: Considered confounders and controlled for less than 60%.
Attrition bias	Withdrawal and drop-out rates Size of missing data	Weak: Follow up participation rate of more than 80% or missing data of less than 20%. Moderate: Follow up participation rate of 60–79% or missing data of 20–40%. Strong: Follow up participation rate of less than 60% or missing data of more than 40%.

**Table A2:** Cross-sectional studies included in review.

Author, Year, and Country	Study Population	Age	Gender	Sample Size (in Analysis)	Smoking	Outcome	Risk of Bias					Results	Adjustment for Other Covariates
							Selection	Performance	Detection	Confounding	Attrition		
Low 2021, USA [40]	Part of the National Ambulatory Medical Care Survey between 2006 and 2015. A random sample of visits to non-federally employed, office-based physicians, community health centers, and advanced practice providers	22.9% aged 18–39 years, 33.8% aged 40–59 years, and 43.3% aged 60 years or older	Both, 59.4% were females	322,092 (191,397 females and 130,695 males)	Current smokers vs. never, past, or unknown smokers	CTS identified based on ICD codes	Moderate	Strong	Moderate	Moderate	Moderate	OR 1.32 (CI 1.07–1.63)	Age, sex, obesity, diabetes, hypothyroidism, and chronic kidney disease
Hashimoto 2020, Japan [44]	A random sample of public servants from town of Obuse	Mean age 69.4 (age range 50–89)	Both, 50% were females	379	Pack-years (+100 packs/year × number of years smoked)	Symptoms and nerve conduction study. Subjects with history of CTS diagnosis or surgery were also defined as prevalent cases	Strong	Moderate	Moderate	Strong	Weak	OR 1.0 (95% CI 1.0–1.0)	Unadjusted
Pramchoo 2020, Thailand [41]	Rubber tappers who were household members of the Pawong Rubber Fund Cooperative in Pawong subdistrict, Mueang district	Mean age 49.8 ± 9.0 for CTS cases and 49.1 ± 11.7 for non-CTS participants	Both, 47.6% were females	534	Smoking (no/yes)	CTS diagnosis based on symptoms + clinical examination	Moderate	Strong	Moderate	Strong	Weak	OR 0.8 (95% CI 0.5–1.3)	Unadjusted
El-Helaly 2017, Egypt [45]	Medical technicians of the King Fahd Hospital clinical laboratory	Mean age 37.2 ± 9.5	Both, 67.4% were females	279	Current smoking (no/yes)	Diagnosis of CTS was based on Kamath and Stothard clinical questionnaire and nerve conduction study	Moderate	Strong	Weak	Moderate	Weak	11.1% of 27 participants with CTS and 7.9% of 252 participants without CTS were current smokers. Estimated OR 1.45 (95% CI 0.40–5.24)	Unadjusted. Pregnant, those with diabetes, hypothyroidism, rheumatoid arthritis or with a history of hand trauma were excluded
Ricco & Signorelli 2017, Italy [34]	Consecutive patients referred to a single occupational health service from 31 meat processing plants	Mean age 37.0 ± 10.6	Both, 45.6% were females	434	Current or past smokers vs. never-smokers	Diagnosis of CTS was based on symptoms, clinical signs, and ultrasonography and/or nerve conduction study	Strong	Strong	Weak	Strong	Weak	OR 1.909 (95% CI 1.107–3.293)	Unadjusted
Hegmann 2016, USA [35]	Employees of manufacturing and food processing, and office workers were recruited from 35 facilities, involving 25 diverse industries, located in the states of Illinois, Utah, Washington, and Wisconsin	Mean age 45.1 ± 9.8 years among CTS cases and 40.3 ± 11.5 years among those without CTS	Both, 59.6% were females	1824	Ever-smokers vs. never-smokers	CTS diagnosis was based on symptoms and nerve conduction study	Moderate	Strong	Weak	Moderate	Moderate	OR 1.24 (95% CI 0.96–1.60)	Sex, body mass index and job strain index
Jung 2016, Korea [39]	Healthy orchardists living in Gyeongsangnam-do who participated in the health promotion program	Mean age 58.9 ± 7.9	Both, 53.8% were females	377	Never, past, and current smokers	Diagnosis of CTS was based on symptoms, clinical signs, and nerve conduction study	Moderate	Moderate	Weak	Strong	Weak	Prevalence of past smoking was 44.1% in participants with CTS and 55.9% in those without CTS. Prevalence of current smoking was 50.9% in participants with CTS and 49.1% in those without CTS. Estimated OR 0.69 (CI 0.40–1.17) for past smoking and 0.90 (CI 0.50–1.63) for current smoking	Unadjusted
Kiani 2014, Iran [42]	Convenience sample of patients with diabetes	Mean age 54.0 ± 13.2 for females and 51.6 ± 16.5 for males	Both, 69% were females	432	Current smoking (no/yes)	Symptoms and clinical examination	Strong	Strong	Moderate	Strong	Weak	2.7% of patients with CTS (N = 37) and 6.6% of those without CTS (N = 395) were current smokers. Estimated OR 0.39 (95% CI 0.05–2.99)	Unadjusted
Eleftheriou 2012, Greece [36]	Occupational population (data entry and processing unit)	45.2 ± 9.46	Both, 83.6% females	461	Ever smokers vs. never smokers	Case definition A: history of CTS diagnosed by physician, including surgery due to CTS. Definition B: definition A + suggestive CTS at clinical examination	Moderate	Strong	Moderate	Moderate	Weak	OR of case definition A for ever smoking 1.99 (1.01–2.54). OR of case definition B for ever smoking 1.69 (1.03–2.76)	Age, sex, keyboard use, and physical activity
Shiri 2011, Finland [37]	General population	30 years or older, mean age 52 years	Both, 48% males	6254	Home interview: (1) current smokers (2) past smokers (3) occasional smokers, (4) never smokers.	Clinical diagnosis. Probable, possible CTS, surgery due to CTS	Weak	Weak	Moderate	Weak	Moderate	OR of possible/probable CTS for current smoking 2.1 (1.4–3.1), for past smoking 1.2 (0.8–1.6) and for ever smoking 1.50 (1.1–2.0) OR of surgery due to CTS for current smoking 1.5 (0.7–3.2)	Age, sex, education, somatization, hand grip with high forces, and work using vibrating tools
Maghsoudipour 2008, Iran [43]	Occupational population (auto factories)	Mean age in CTS group 29.85, years mean age in healthy group 27.95 years	Both, 23% were females	395	Cigarette smokers vs. nonsmokers	Symptoms + clinical diagnosis + nerve conduction study	Moderate	Strong	Weak	Weak	Weak	OR 4.68 (95% CI 1.08–11.80) for current smoking	Age, gender, marital status, body mass index, education, job duration, workplace risk factors (force exertion > 1 kg, rapid hand movement, break time > 75 min, wrist bending/twisting, job rotation, using vibrating tools
Atroshi 2007, Sweden [10]	General population	25–65	Both, 53.8% females	2003 (925 males and 1078 females)	Current smokers versus non-smokers	Symptoms + clinical diagnosis + nerve conduction study	Weak	Strong	Weak	Moderate	Weak	OR 1.79 (95% CI 1.10–2.90)	Sex, age ≥ 40 years, overweight and keyboard use ≥1 h/day
Frost 1998 Denmark [38]	Occupational population (slaughterhouse workers and chemical factory workers)	Mean age 40.5 years	Both, 84.7% were males	1141 (966 males and 175 females)	Ever smokers	Symptoms, clinical diagnosis, nerve conduction study or previous surgery due to CTS	Moderate	Strong	Weak	Weak	Weak	OR for ever smoking 0.65 (95% CI 0.34–1.24)	Age (stratified), gender, occupational risk factor, wrist trauma, body mass index, and medical condition

**Table A3:** Case-control studies included in the review.

Author, Year and Country	Study Population	Age	Sex	Sample Size (in Analysis)	Smoking	Outcome	Risk of Bias					Results	Adjustment for Other Covariates
							Selection	Performance	Detection	Confounding	Attrition		
Ulbrichtova 2020, Slovakia [46]	Cases were consecutive electrophysi- ologically confirmed CTS patients and controls were a randomly selected patients without any known systemic disease or symptoms of CTS who were treated at the Clinic of Occupational Medicine and Toxicology	Age range 27‒63 for cases and 21–63 for controls, mean age 52.5 ± 5.9 for cases and 49.6 ± 9 for controls	Both, 51.9% of cases and 54% of controls were females	162 cases and 300 controls	Never/past/current.	Symptoms and nerve conduction study	Weak	Moderate	Weak	Moderate	Weak	OR 1.51 (95% CI 0.94–2.42) for smoking; It seems the OR is for ever vs. never smoking	Age, sex, body mass index, alcohol drinking, diabetes, and hypertension
Bhanderi 2017, India [47]	CTS cases were patients managed at K M Patel School of Physiotherapy, Gujarat. Controls were patients attending the same institute, patients attending other outpatient departments or relatives of patients	Mean age 47.6 ± 10.96 years (range, 18–80) for cases and 47.5 ± 10.89 (range, 20–80) for controls	both, 78.8% were females	137 cases and 274 controls	Never, past, and current smokers	Symptoms, clinical, and nerve conduction study	Moderate	Moderate	Weak	Moderate	Weak	OR 0.37 (CI 0.02–6.17) for past smokers and 1.40 (CI 0.71–2.78) for current smokers	Education, family history, short stature, obesity, diabetes, rheumatoid arthritis, hypothyroidism, hypertension, and computer use
Guan 2018, China [48]	Cases were outpatient and surgical CTS cases free of other diseases recruited from a single medical center and controls were outpatients	41–70	Both, 82.5% of cases and 82.5% of controls were females	1512 cases and 4536 controls	Current smokers vs. nonsmokers	Symptoms, clinical, and nerve conduction study	Moderate	Strong	Weak	Strong	Weak	OR 4.86 (95% CI 3.99–5.73)	Matched by sex
Coggon 2013, UK [49]	Cases were CTS patients and controls were patients attended the accident and emergency department	20–64	Both, 68% were females	1230 (457 cases and 773 controls)	Never, past, and current smokers	Symptoms + nerve conduction study	High	Moderate	Low	Low	Low	OR 1.1 (CI 0.8–1.4) for past smokers and 0.6 (CI 0.4–0.8) for current smokers	Age, sex, ethnicity, body mass index, mental health, repeated movements of wrist or fingers, using hand-held vibrating tools, supervisor or colleagues support, and little choice in how or what work is done or in timetable and breaks
Mattioli 2009, Italy [50]	Cases: random sample of local hospitals. Controls: random sample of national health service registries	18–65 years	Both, 84% were females	191 cases and 286 controls.	Never-smokers, past smokers, current smokers, and pack-years	Surgery due to CTS (symptoms, clinical diagnosis, and nerve conduction study)	Weak	Weak	Weak	Moderate	Weak	OR 0.7 (95% CI 0.4–1.1) for past smoking and 1.1 (95% CI 0.7–1.7) for current smoking	Frequency matching by age and gender
Fung 2007, Hong Kong [51]	Outpatient CTS patienta and patient controls were recruited from three centers	Age range 18–60, mean age 46.3 ± 9.1	Both, 84.5% were females	166 cases and 111 controls	Current smokers vs. non-smokers	Symptoms + clinical assessment and nerve conduction study for atypical cases (51% of cases)	Moderate	Strong	Weak	Moderate	Weak	4.2% of cases and 16.2% of controls were smokers; OR for smoking 0.23 (0.09–0.57)	Unadjusted Patients with rheumatoid arthritis, diabetes, hypothyroidism, cervical spondylosis, post-traumatic wrist deformities, and pregnant women were excluded from both cases and controls
Geoghegan 2004, UK [11]	General practice population, the West Midland section of The UK General Practice Research Database	16–96, Mean age 46	Both, 72% were females	16955 (3391 cases and 13564 controls)	Current smokers vs. non-smokers	Registry data: diagnosis of CTS, surgery due to CTS	Weak	Strong	Moderate	Moderate	Strong	OR of CTS was 1.03 (CI 0.93–1.13) for smoking; OR of surgery due to CTS was 1.04 (CI 0.86–1.26)	Age, sex, general practice, date of diagnosis, and mean annual consultation rates
Karpitskaya 2002, USA [52]	Patient population. Patients who underwent CTR, control group formed of patient seen for general reconstructive surgery or those with acute hand diagnoses	Mean age 50 ± 15 for cases, 47 ± 14 for controls	Both, 59.6% were females	514 cases and 100 hospital controls	Never, past, and current smoking, and pack-years, estimates reported for current smokers vs. non-smokers	Surgery, due to CTS based on hospital records	Moderate	Weak	Weak	Strong	Weak	26.3% of cases and 33% of controls were smokers; OR 0.72 (95% CI 0.45–1.15) for current smoking	Unadjusted
Ferry 2000, UK [53]	General practice population. The Royal College of General Practitioners’ Oral Contraception Study attendees	Mean age 41.9 for both groups	Female	1264 cases and 1264 controls	Smokers vs. non-smokers	General practitioner diagnosed CTS	Weak	Strong	Moderate	Moderate	Weak	OR 1.05 (95% CI 0.89–1.23)	Age
Wieslander 1989, Sweden [54]	Patients undergoing CTR as cases and other surgical patients as control group 1 and population sample as control group 2	Age range 20–66	Males	177 (34 cases and 143 controls), two hospital controls and two population controls for each case	Current smokers vs. non-smokers	Surgery due to CTS (clinical diagnosis + nerve conduction study)	Moderate	Strong	Weak	Moderate	Weak	OR for current smoking 1.5 (0.7–3.5) for cases and all controls	Age and year of operation for hospital controls

**Table A4:** Cohort studies included in the review.

Author, Year and Country	Study Population	Age	Gender	Sample Size (in Analysis)	Smoking	Outcome	Risk of Bias					Results	Adjustment for Other Covariates
							Selection	Performance	Detection	Confounding	Attrition		
Rydberg 2020, Sweden [25]	A population-based study of the Malmö Diet and Cancer Study, median follow-up 21.4 years	46–73 mean 57 ± 7.6	both, 60% were females	30,323	Current smoking, yes/no	Information on diagnosis of CTS was obtained from register data, surgical codes were not avail-able; only ICD codes for clinical, and hospital-based CTS were available	Weak	Strong	Moderate	Moderate	Weak	HR 1.06 (CI 0.92–1.23)	Age, sex, alcohol consumption, body mass index, hypertension, and the use of antihypertensive treatment
Hulkkonen 2020, Finland [26]	The Northern Finland Birth Cohort 1966 participants, mean follow-up time 18.3 years	31 years	Both, 48.5% were females	6326 (3260 males, 3066 females)	Past or current smokers vs. never smokers	Diagnosis of CTS was based on out- and inpatient specialist care register data	Moderate	Strong	Moderate	Weak	Weak	HR 1.48 1.12–1.96) for both sexes combined and 1.66 (1.19–2.32) for females. The HR was not significant for males	Sex, occupational class, body mass index, exposure to heat, exposure to temperature changes, and exposure to vibration (for both sexes combined only)
Hulkkonen 2019, Finland [58]	The Northern Finland Birth Cohort 1966 participants, mean follow-up time 18.3 years	31 years	Both, 52.2% were females	8703 (4156 males, 4547 females)	Number of pack-years	Diagnosis of CTS was based on register data on out- and inpatient specialist care	Weak	Moderate	Moderate	Moderate	Weak	HR was 0.94 (CI 0.52–1.71) for packyears ≤10 and was 1.89 (CI 1.14–3.12) for pack-years >10 for males. It was 1.54 (1.11–2.15) for packyears ≤10 and 1.90 (CI 1.20–3.01) for pack-years >10 for females	Body mass index, socioeconomic status, and diabetes
Pourmemari 2018, Finland [27]	Population-based study linked to the Hospital Discharge Register for specialist medical care, 11-year follow-up	52 ± 14 years	Both, 54% were females	6177	Never/occasional/past/current smoking	Register data on carpal tunnel release	Weak	Moderate	Weak	Moderate	Weak	HR 1.2 (CI 0.5–2.9 for male current smokers, 1.0 (CI 0.6–1.7 for female current smokers and 1.1 (CI 0.7–1.7) for both sexes combined current smokers. HR 1.1 (0.5–2.7) for male past smokers, 1.3 (0.7–2.3) for female past smokers and 1.2 (0.8–1.9) for both sexes combined past smokers	Age and sex
Harris-Adamson 2013, USA [55]	Full-time workers in industries primarily engaged in manufacturing, production, service, and construction	31% were <30 years, 24% were 30–39 years, 26% were 40–49 years and 19% were 50 years or older	Both, 47% were females	3514	Never, past, current	CTS diagnosis based on symptoms and nerve conduction study	Weak	Moderate	Weak	Strong	Weak	IRR 1.09 (0.78–1.51) for current smokers and 1.05 (0.70–1.54) for past smokers	Unadjusted
Gell 2005, USA [22]	Workers from four industrial and three clerical worksites, 5.4 years follow-up	19–69	Both, 71% females	432	Smokers vs. non-smokers	Symptoms, clinical diagnosis and nerve conduction study or self-reported surgery due to CTS, since the time of the initial screening	Moderate	Strong	Weak	Strong	Strong	OR for smoking 0.88 (0.37–2.03)	Unadjusted
Werner 2005, USA [56]	Workers from an automobile assembly plant, 1-year follow-up	Mean age 49.8 for participants with CTS and 47.5 for those without CTS	Both, 25.5% were females	189	Currently smoking (no/yes)	Symptoms + nerve conduction study or self-reported physician diagnosed CTS, since the time of the initial screening	Strong	Strong	Weak	Strong	Weak	56% of 20 participants with CTS and 51% of 169 participants without CTS during the follow-up were smokers at baseline, estimated risk ratio 1.08 (95% CI 0.71- 1.65)	Unadjusted
Nathan 2002, USA [57]	Four industrial sites (a steel mill, meat/food packaging, electronics, and plastics), 11-year follow-up	Mean age 34.86 ± 9.96	Both, 56.6% were males	256 (145 males and 111 females)	Smokers vs. non-smokers, a retrospective data	Symptoms + nerve conduction study or surgery due to CTS since the last follow-up visit	Moderate	Strong	Weak	Weak	Strong	Smokers vs. non-smokers, OR = 2.42 (1.06–5.51)	Age, gender, body mass index, vibration, and endocrine condition
Nathan 2005, USA [23]	17-year follow-up		60% males	148	Sum of the ratings of current smoking in 1984, 1989, and 1994 to 1995, where smoking equalled 1 and non-smoking equalled 0	As above						Current smoking vs. non-smoking OR = 1.22 *p* = 0.66. Confidence interval not reported.	Gender, age, body mass index, repetition, heavy lifting, keyboard use, vibration, and force
Roquelaure 2001 France [24]	Occupational population, five footwear factories	Mean age 40.7 ± 7.7	Both, 61% were females	134	Current smokers vs. non-smokers	Clinical diagnosis	Moderate	Strong	Moderate	Strong	Moderate	OR for current smoking 0.5 (0.1–2.2)	Unadjusted
